# Bibliometrics Analysis of Butyrophilins as Immune Regulators [1992–2019] and Implications for Cancer Prognosis

**DOI:** 10.3389/fimmu.2020.01187

**Published:** 2020-06-30

**Authors:** Yixi Wang, Na Zhao, Xianwen Zhang, Zhenhua Li, Zheng Liang, Jinrong Yang, Xingyu Liu, Yangzhe Wu, Kebing Chen, Yunfei Gao, Zhinan Yin, Xuejia Lin, Haibo Zhou, Dongbo Tian, Yang Cao, Jianlei Hao

**Affiliations:** ^1^Guangdong Provincial Key Laboratory of Stomatology, Guanghua School of Stomatology, Hospital of Stomatology, Sun Yat-sen University, Guangzhou, China; ^2^Department of General Surgery, Tianjin Medical University General Hospital, Tianjin Medical University, Tianjin, China; ^3^School of Basic Medical Sciences, Chengdu University of Traditional Chinese Medicine, Chengdu, China; ^4^The Sixth Affiliated Hospital of Guangzhou Medical University, Qingyuan People's Hospital, Qingyuan, China; ^5^Zhuhai Precision Medical Center, Zhuhai People's Hospital, Zhuhai Hospital Affiliated With Jinan University, Jinan University, Zhuhai, China; ^6^Faculty of Medical Science, The Biomedical Translational Research Institute, Jinan University, Guangzhou, China; ^7^Department of Orthodontics, Changsha Stomatological Hospital, Changsha, China; ^8^Guangdong Provincial Key Laboratory of Bone and Joint Degeneration Disease, The Third Affiliated Hospital of Southern Medical University, Guangzhou, China

**Keywords:** butyrophilin, γδ T cells, bibliometrics, VOSviewer, lung cancer, breast cancer

## Abstract

The butyrophilins (BTNs) represent a unique family of immunoglobulin. They were considered to be involved in milk lactation after their discovery in 1981. With the development of research, an increasing number of research revealed that BTNs play important roles in immune regulation [1992–2019]. Our research aimed to summarize the BTN research status and their relationship with lung cancers and breast cancers by bibliometrics and bioinformatics methods. Our results indicate that the researches on immune-regulatory functions of BTNs gradually developed from 1992 to 2006, whereas they increased quickly after 2007. There are international cooperations among 56 countries, of which the United States is the most active one with the highest number of studies as well as highest citations. By coauthorship and cocitation analysis, we showed that Adrian Hayday, who is active in γδ T-cell field, was an active author in BTN publications with average year of 2015 and led a subfield. By keywords co-occurrence analysis, we found that γδ T cell, which is an important cancer immune regulator, is one important hotspot. Finally, we found that several BTN members' expression levels were significantly correlated with prognosis of lung cancer and breast cancer patients. Thus, these BTNs might play immune regulatory effects and could serve as potential biomarkers for cancer.

## Introduction

The modulatory effects of T-cell activation are significantly mediated by costimulatory molecules expressing on antigen-presenting cells. Recent discoveries show that another superfamily of immunoglobulin, the butyrophilin (BTN) family, which is similar to B7 family, has been involved in immune modulation (1). Initially, the BTN, which was discovered in 1981, was found in milk-secreting epithelial cells and constituted the milk protein (2, 3). With the identification of additional members of this family, increasing evidence shows that BTNs play roles in immune regulation (4). To date, the human BTN superfamily has been found to include 7 BTN genes (*BTN1A1, BTN2A1, BTN2A2, BTN2A3, BTN3A1, BTN3A2, BTN3A3*), 5 BTN-like genes (*BTNL2, BTNL3, BTNL8, BTNL9, BTNL10*), and the SKINT-like factor (*SKINTL*). Twenty-one genes have been found in mice in this superfamily: *Btn1a1, Btn2a2, Btnl1, Btnl2, Btnl4, Btnl5, Btnl6, Btnl7, Btnl9, Btnl10*, and *Skint1, Skint2, Skint3-11* (1). Growing numbers of researches have shown that BTNs play a role in autoimmune diseases (5), infections (6), metabolic disorders (7), and cancers (8, 9) through immune stimulation and inhibition.

Despite many advances in understanding the role of BTNs, there is still a lack of global and comprehensive report that helps researchers to get a quick overview and find meaningful research directions. Bibliometrics analysis is defined to analyze the open publications in a statistical way that could summarize the current research status (10, 11) and foresee future trends quantitatively and qualitatively (12). In this research, we aimed to analyze the progression of BTN researches with the visualization techniques, VOSviewer, based on bibliometrics method and explore the relationships between BTN family members and cancers.

Members of BTN family serve as important regulators of different T-cell subsets, especially the γδ T cells, in humans and mice (4, 13, 14). Different BTN family members can stimulate corresponding γδ T-cell subsets potentially through interaction with specific T cell receptor (TCR). Previous research revealed that BTN3A1 was required in the activation of human Vγ9Vδ2 T cells (15), and BTNL3 and BTNL8 were found to bind Vγ4^+^ TCR (16, 17). In mice, Skint1, the new member of BTN family, was required for positive selection of Vγ5^+^ T cells in the embryonic thymus and contributed to normal levels of these dendritic epidermal T cells in skin, which play an important role in wound healing and preventing cancer (18, 19). BTNL1 and BTNL6 jointly regulated intestinal Vγ7 γδ T-cell function in mice, and human gut epithelial cells expressed BTNL3 and BTNL8 and stimulated Vγ4 γδ T cells (17). Moreover, γδ T cells represent a strong protective factor among leukocytes, which correlates with better prognosis of cancer (20). Collectively, these results let us hypothesize that BTNs in tumor microenvironment regulate γδ T-cell functions and serve as biomarkers for cancer prognosis.

Lung cancer is the top one cause of cancer deaths worldwide, leading to 1.6 million deaths every year (21–23). Non-small cell lung cancer (NSCLC) is the most common type of lung cancer. Lung adenocarcinoma (LUAD) and lung squamous cell carcinoma (LUSC) are two major subtypes of NSCLC, which accounts for almost 85% of cases (23). Programmed death protein 1 (PD-1) and programmed death ligand 1 (PD-L1), the two famous members of B7 family, were found to be important immune checkpoints and immunotherapy, which aimed to produce encouraging responses for NSCLC by reversing their expression levels (24–26). However, the usage of PD-1/PD-L1 inhibitors also has limitations and reality that most patients still do not benefit from their use (27). Moreover, a clinical trial showed that PD-L1 expression was not predictive of good outcomes that low expression could also observe benefit from its inhibitor treatment (28). This might be because the interaction between tumor microenvironment and immune system also involves other regulators apart from PD-1/PD-L1 axis. Thus, identification of novel predictive biomarkers and targets for therapy is important. Genome-wide analysis revealed the association between encoding BTNL2, a member of BTN family, and LUAD (29–31). Considering the TCR-specific regulatory effects of BTN family members on γδ T cells, which play critical roles in cancer, our research aimed to evaluate the prognostic values of BTNs in LUAD and LUSC, as well as breast cancer.

In this research, we summarized publications of BTNs and analyzed the publication amount, citation, coauthorship, and trends of research area. Additionally, we analyzed the expression of every BTN member in LUAD, LUSC, and breast cancer. Several BTN members showed significant correlation with overall survival (OS). These results suggested a functional role of BTNs in tumor immunity.

## Methods

### Database and Search Design

We utilized Scopus (Elsevier, Amsterdam, The Netherlands), the largest abstract database of keyword searching and citation analysis coverage, as our main database (32–34). The following searching words: TITLE-ABS-KEY (“butyrophilin^*^”) AND PUBYEAR > 1979 AND PUBYEAR <2020 were used in Scopus. The database search was done on a single day, January 10, 2020, so as to avoid significant fluctuations in citations, as well as numbers of studies. Four hundred thirty-five studies were acquired through this step.

Titles, abstracts, and keywords of these 435 studies were screened and infiltrated manually. Full texts were further checked if necessary. The inclusion criteria were as follows: (1) a clear correlation with BTN, (2) focus on human or mice subjects, (3) not editorial notice, such as erratum, and (4) focus on immune modulation. In total, 260 studies were included after filtration by two authors independently. The publication type, annual publication numbers, and total citations were analyzed. The publication list (of the 260 articles in csv file) was used for bibliometrics analysis, and xls file is shown in the Supplementary Table 1.

### Analysis With VOSviewer

The csv file containing these 260 studies on immune modulation in human or mouse was imported to VOSviewer to perform coauthorship, co-occurrence, and cocitation analysis.

#### Country Coauthorship

Country coauthorship was performed by VOSviewer in coauthorship analysis in the unit of countries. Circle labels were used to represent the country elements, which were analyzed. The areas of the circles (not the diameter) were proportional to the numbers of total publication citations of each country. The minimum number of total citations of a country was set to 4 to be shown.

#### Author Coauthorship

Author coauthorship was performed by VOSviewer in coauthorship analysis in the unit of authors. The circle labels represented different authors, and their area showed the total citations of each author. The minimum number of citations of an author was set to 4 to be shown.

#### Author Cocitation

Author cocitation was performed by VOSviewer in cocitation analysis in the unit of cited authors. The minimum number of citations of an author was set to 50 to be shown.

#### Author Keywords Co-occurrence Analysis

We uniformed “γδ T cells, γδT cells, gamma delta T cell, gamma delta T cells, and gamma delta T lymphocyte” to “γδ T cell.” In addition, “butyrophilin-like 2” was uniformed to “btnl2” when “btnl2” was not found in the author keywords of the same publication. “Butyrophilins” was uniformed to “butyrophilin.” “Butyrophilin 3a1” was uniformed to “btn3a1” when “btn3a1” was not found in the author keywords of the same publication. “T cells” and “T lymphocytes” were uniformed to “T cell.” Then the csv file was imported to VOSviewer to perform co-occurrence analysis in the unit of author keywords. The minimum number of occurrences of a keyword was set to 2 to be shown. Total strength of the co-occurrence links with other keywords was calculated.

### Estimation of Prognostic Values of BTN Family Members on LUAD and LUSC

The Kaplan–Meier plotter (http://kmplot.com/analysis/) was employed to perform the survival analysis of BTN family members with gene chip data in 719 patients with LUAD, 524 patients with LUSC, and 3,951 patients with breast cancer. The plotter separated patients into high and low expression groups according to the gene transcription level of each specific gene and created Kaplan–Meier plots. In the meantime, the hazard ratio (HR) with the 95% confidence interval and the log-rank *p*-value were calculated and marked on the chart. The numbers at risk of each group at different time points are displayed under the curves. When *p*-values were below 0.05, they were considered statistically significant.

## Results

### The Publication Time and Citation of the Studies of BTNs on Immune Modulation

**Figure 1:**
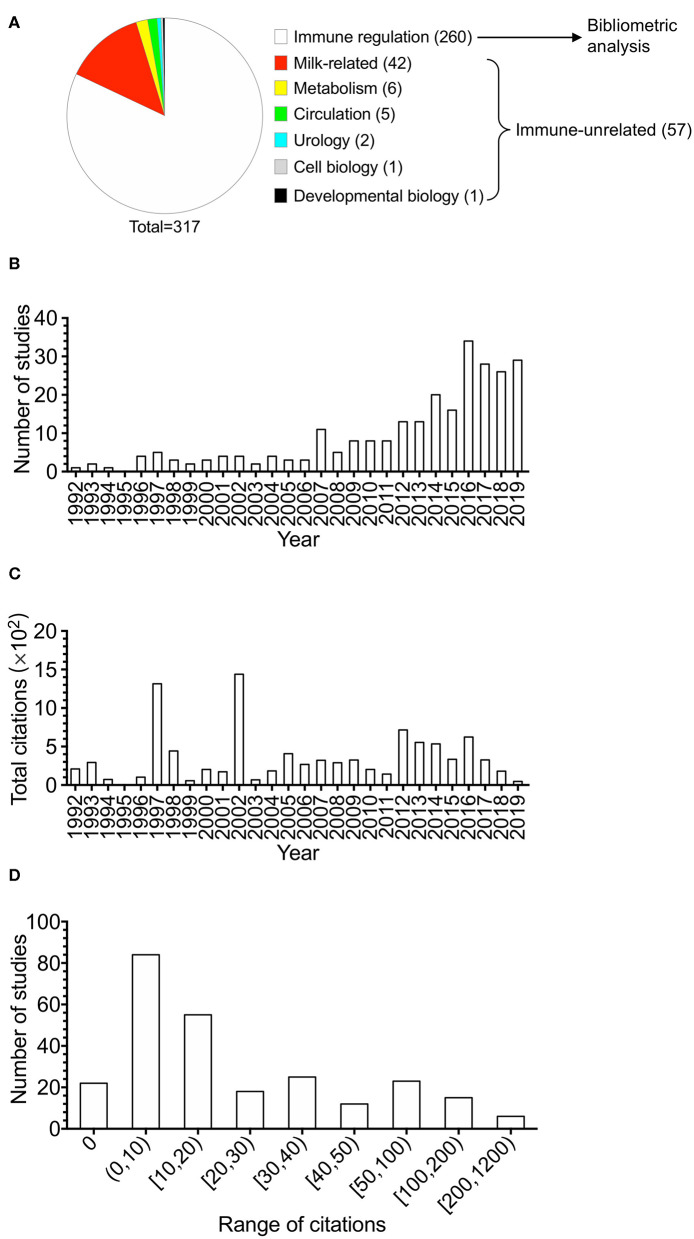
The overview of the studies of butyrophilins on immune modulation. Studies in butyrophilins were acquired from Scopus. **(A)** The composition of the 317 studies acquired from Scopus studying human and mouse butyrophilins is shown. **(B)** The number of studies of immune-related butyrophilins for each year (from 1992 to 2019) was calculated and shown. **(C)** Annual total citations of studies every year was calculated and shown. **(D)** The article citation distributions were calculated and shown. The ranges of citation numbers were divided into 0, 0–10, 10–20, 20–30, 30–40, 40–50, 50–100, 100–200, and 200–1,200.

With the interest of human and mouse studies, the composition of the 317 studies is shown in Figure 1A. Studies on immune regulation in human or mouse accounted for 81.8% (260 studies). Immune-unrelated studies accounted for 18.0% (57 studies), including milk-related studies, studies on metabolism, circulation, urology, cell biology, and developmental biology. The publications (totally 260 studies, listed in the Supplementary Table 1) of BTNs in immune regulation in human or mouse were utilized for the following analysis. For the publication types of the 260 studies, research articles accounted for 74% (192 studies), which were the majority. Reviews accounted for 17% (45 studies), which were the second largest part. The remaining 9% were other types of documents, including book chapter (2%, five studies), notes (2%, six studies), short surveys (2%, five studies), letters (1%, three studies), and conference papers (2%, four studies).

Annual number of studies is shown in Figure 1B. Although the time period we searched was from 1980 to 2019, the research of BTNs in immune modulation did not arise until 1992. Generally, the annual publications could be divided into two time stages. The researches on BTNs on immune modulation increased slowly in 1992–2006, whereas they increased quickly after 2007 and reached a peak of publications in 2016.

Total citations of all BTN studies on immune modulation had fluctuations over the years (Figure 1C). In the first stage, two obvious peaks in 1997–2002 are shown with two highly cited articles in each year (35, 36). Total citations increased slowly during the second stage when total publications increased quickly, considering the fact that recently published papers need time to accumulate citations.

The average citation of all the studies was 37.77 times through dividing the total citation times (9,820) by 260. Figure 1D demonstrates the distributions of the number of citations of different ranges. One hundred sixty-one studies accounting for 61.9% were cited fewer than 20 times, of which 22 studies were not cited by any other studies. Twenty-one studies received more than 100 citations, among which the highest citation was 1,190. Among the top 15 highly cited articles, four studies were about γδ T cells (15, 18, 37, 38), including one paper about epidermal γδ T cells (18). The 15 top-cited articles are shown in Table 1.

**Table 1:** Top-cited articles for butyrophilins as immune regulators.

**Rank**	**Journal**	**IF**	**NA**	**NC**	**Citations per article**	**Article title**	**Year**	**Authors**
1	*Nature Genetics*	25.455	4	1,702	1,069	A candidate gene for familial Mediterranean fever	1997	Bernot A, et al.
					353	Sarcoidosis is associated with a truncating splice site mutation in BTNL2	2005	Valentonyte R, et al.
					166	Skint1, the prototype of a newly identified immunoglobulin superfamily gene cluster, positively selects epidermal γδ T cells	2008	Boyden LM, et al.
					114	A genome-wide association study identifies two new susceptibility loci for lung adenocarcinoma in the Japanese population	2012	Shiraishi K, et al.
2	*Nature Reviews Immunology*	44.019	1	1,190	1,190	The B7-CD28 superfamily	2002	Sharpe AH, et al.
3	*Immunity*	21.522	2	336	170	The intracellular B30.2 domain of butyrophilin 3A1 binds phosphoantigens to mediate activation of human Vγ9Vδ2T Cells	2014	Sandstrom A, et al.
					166	Coinhibitory pathways in the B7-CD28 ligand–receptor family	2016	Schildberg FA, et al.
4	*Lancet*	59.102	1	227	227	Role of human-milk lactadherin in protection against symptomatic rotavirus infection	1998	Newburg DS, et al.
5	*Blood*	16.601	1	217	217	Key implication of CD277/butyrophilin-3 (BTN3A) in cellular stress sensing by a major human γδ T-cell subset	2012	Harly C, et al.
6	*Journal of Neuroscience Research*	4.139	1	209	209	Myelin/oligodendrocyte glycoprotein is a unique member of the immunoglobulin superfamily	1992	Gardinier MV, et al.
7	*Nature Immunology*	23.530	1	198	198	Butyrophilin 3A1 binds phosphorylated antigens and stimulates human γδ T cells	2013	Vavassori S, et al.
8	*Proceedings of the National Academy of Sciences of the United States of America*	9.580	1	180	180	Myelin/oligodendrocyte glycoprotein is a member of a subset of the immunoglobulin superfamily encoded within the major histocompatibility complex	1993	Pham-Dinh D, et al.
9	*Journal of Neuroimmunology*	2.832	1	145	145	Antibodies to neuron-specific antigens in children with autism: possible cross-reaction with encephalitogenic proteins from milk, *Chlamydia pneumoniae* and *Streptococcus* group A	2002	Vojdani A, et al.
10	*Journal of Immunology*	4.718	1	126	126	BTNL2, a butyrophilin-like molecule that functions to inhibit T-cell activation	2006	Nguyen T, et al.
11	*Journal of Molecular Evolution*	1.782	1	113	113	Evolutionary study of multigenic families mapping close to the human major histocompatibility complex class I region	1993	Vernet C, et al.

*NA, number of highly cited articles; NC, number of citations; IF, 2018 impact factor; Rank, rank by NC*.

### The Coauthorship Analysis in the Unit of Countries

The 260 articles, studying BTNs in human and mouse, included authors from 56 countries in total. With the minimum number of documents and citations of a country was set to 4; 18 countries of these 56 countries met the thresholds.

**Figure 2:**
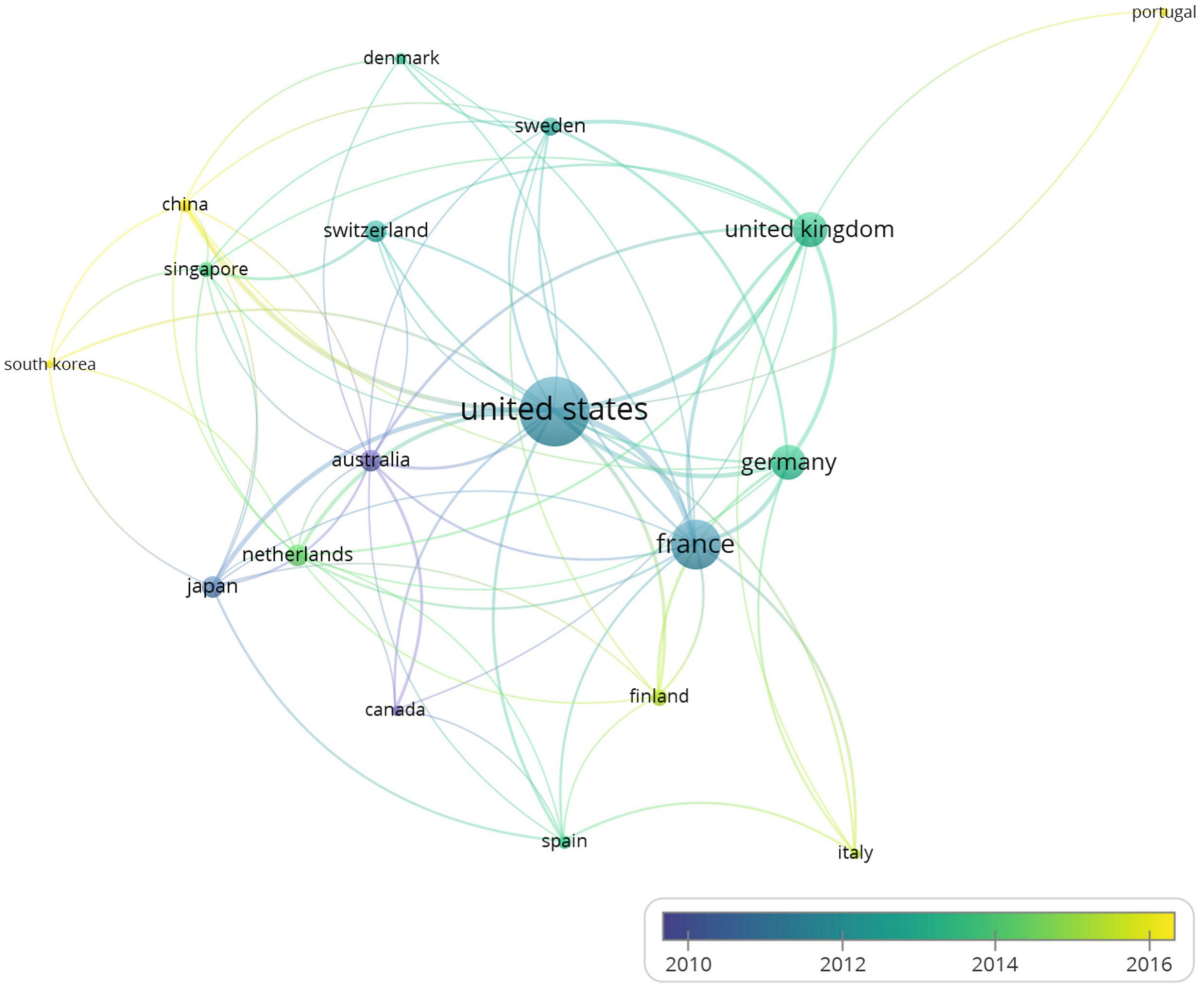
The overlay visualization map of country coauthorship analysis. The 260 studies on immune modulation in human and mouse (listed in the Supplementary Table 1) were analyzed by VOSviewer, and overlay visualization map was shown. Countries were represented by circle labels. The distance between two circles indicated the relatedness of their link. The strength of the coauthor link between two countries was represented by the thickness of the connecting curve lines. The area of the circle was determined by the numbers of the total citations contained by each country. Different colors of the circles indicated the average year of the studies according to the bar on the lower right corner. Color range indicated average year of publications in each country.

The overlay visualization map (Figure 2) of country coauthorship analysis showed the coauthorship of the 18 countries, which indicated close cooperation among different countries. The United States has the highest total citations and has cooperation relationships with many countries. The map also reveals that China and South Korea are countries that started working on the immune-related functions of BTNs more recently than other countries.

### Coauthorship Analysis and Cocitation Analysis

**Figure 3:**
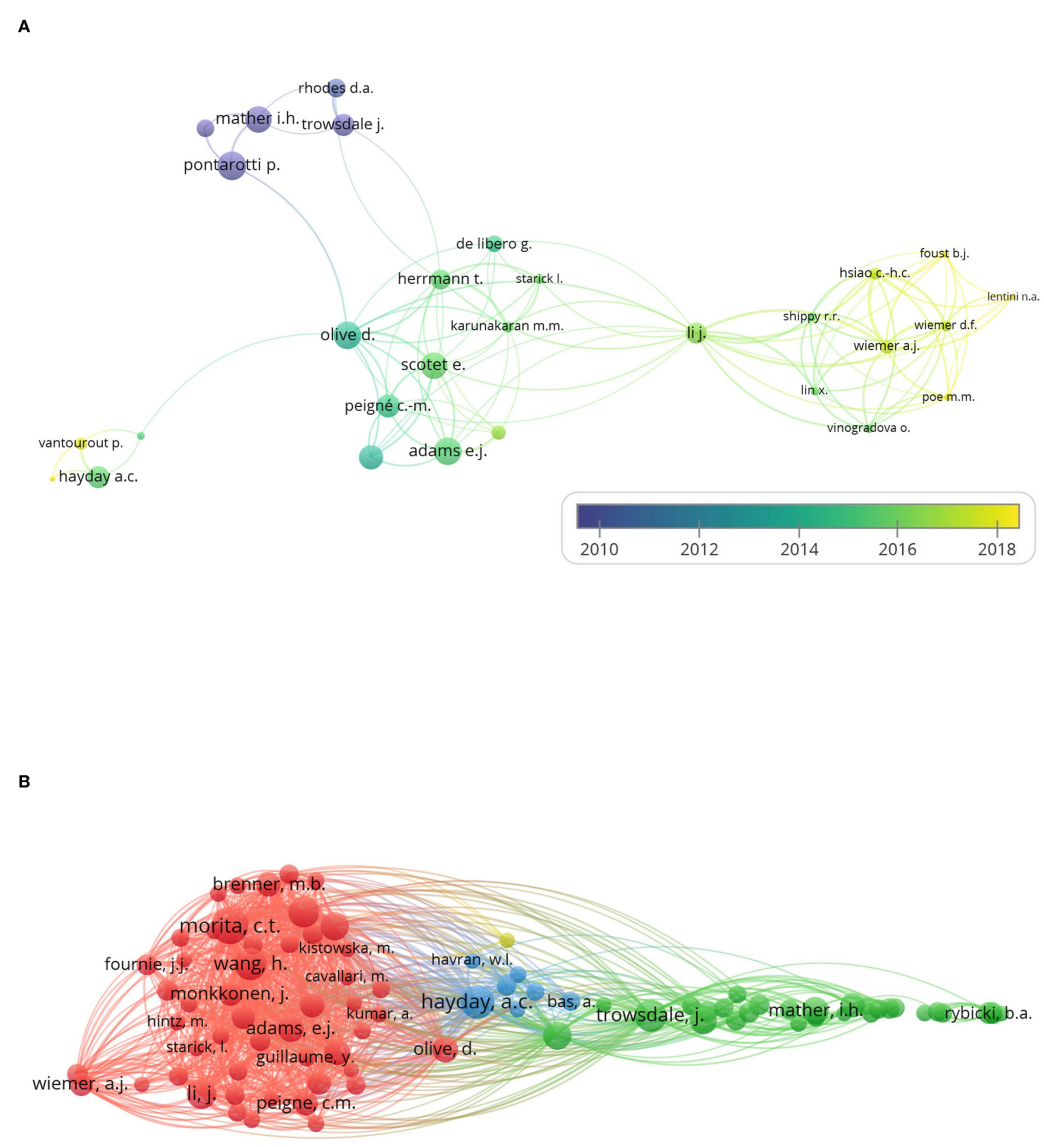
Coauthorship analysis and cocitation analysis. The 260 studies on immune modulation in human and mouse (listed in the Supplementary Table 1) were analyzed by VOSviewer, and coauthorship analysis and cocitation analysis are shown. **(A)** The overlay visualization map of author coauthorship analysis. The area of the circle was determined by total citations of each author. Colors of the circles indicated the average year of the studies according to the symbol on the lower right corner. The distance between circles indicated their relatedness. The strength of the coauthor link between two countries was represented by the thickness of the connecting curve lines. **(B)** The network visualization map of author cocitation analysis. Different colors of the circles represented clusters divided by cocitations. The area of circles indicated the total citation number of each author.

With the minimum number of documents and citations of an author was set to 4, and 34 authors met the thresholds. Five authors of them had no linkage with others. The remaining 29 authors are shown in the map (Figure 3A).

The overlay visualization map (Figure 3A) of author coauthorship analysis showed the cooperation among authors, the total citations of each author, and the average year of their publications. It revealed that Adrian Hayday was among the authors who published studies on the average year of 2015, suggesting an active researcher in BTN field.

As for the cocitation analysis, 87 authors met the threshold with the minimum number of citations of an author set to 50. The network visualization map (Figure 3B) of author cocitation analysis through cited authors showed the similarities between different studies and the influence of authors.

### Keywords Co-occurrence Analysis

**Figure 4:**
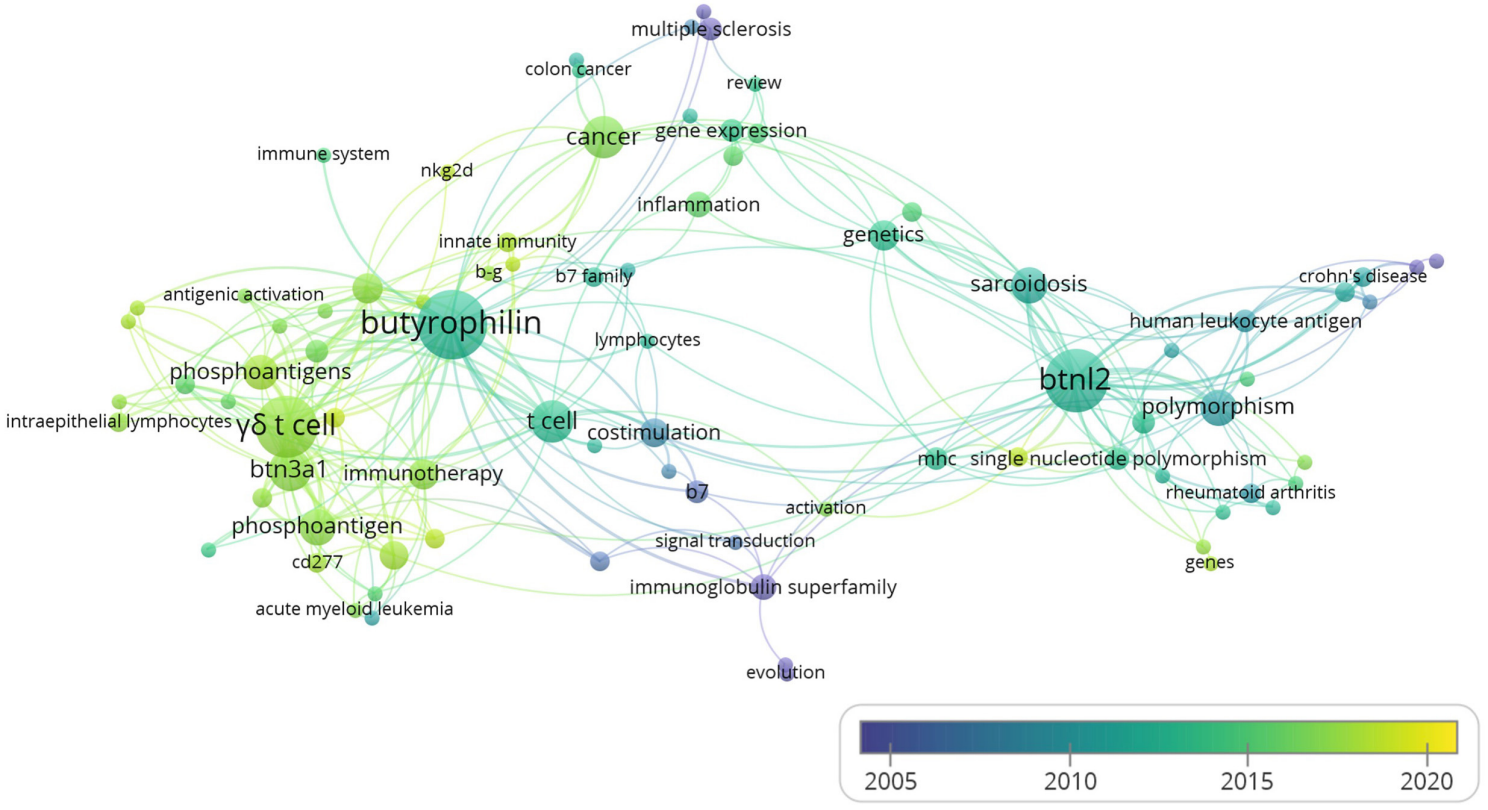
The overlay visualization map of author keywords co-occurrence analysis. Figure was generated by VOSviewer from the data of the 260 studies on immune modulation in human or mouse (listed in the Supplementary Table 1). The area of the circle was determined by occurrences of each keyword. Colors of the circles indicated the average year of the keyword occurrences according to the symbol on the lower right corner.

When the minimum number of occurrences of a keyword was set to 2, 90 keywords met the threshold among the 541 keywords in total. Five keywords of them had no linkage with others. The remaining 85 keywords are shown in the map. The overlay visualization map scaled by occurrences (Figure 4) showed the hotspots in the field of immune modulations exerted by BTNs. As depicted in the chart, “γδ T cell” ranked one of the top keywords with the second most occurrence with the average publication year of 2017, suggesting a new hotspot in BTN field. Although “Btnl2” has significant occurrence, with average publication year of 2012, it is inclusive in BTN family. Of note, “costimulation” represents an important keyword that appeared with BTNs, with the average publication year of 2008. This is consistent with the important discoveries that emphasized the regulatory function of BTNs for αβ T cells (4, 14).

### The Association Between BTN Levels and Prognosis of Lung Cancer and Breast Cancer

**Figure 5:**
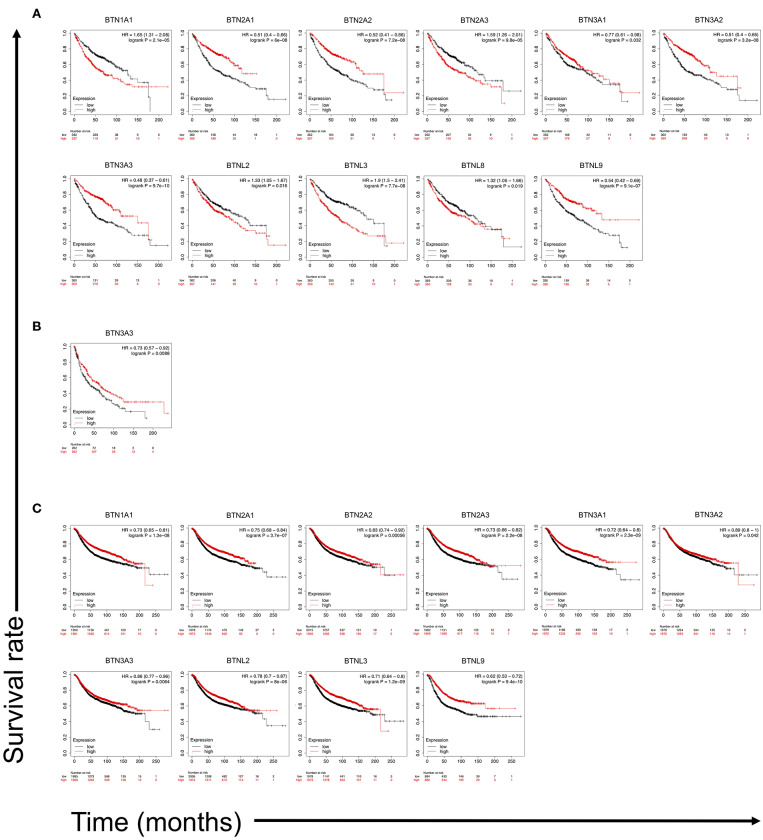
Kaplan–Meier overall survival (OS) survival curves of LUAD and LUSC cases according to gene expression levels of butyrophilin family members. The Kaplan–Meier plotter (http://kmplot.com/analysis/) was employed to calculate survival of butyrophilin family members expression high vs. low in gene chip data on LUAD (*n* = 719), LUSC (*n* = 524), and breast cancer (*n* = 3,951) patients. **(A)** Overall survival curves of LUAD patients based on BTN1A1, BTN2A1, BTN2A2, BTN2A3, BTN3A1, BTN3A2, BTN3A3, BTNL2, BTNL3, BTNL8, and BTNL9 expression levels. **(B)** Overall survival curve of LUSC patients based on BTN3A3 expression levels. **(C)** Overall survival curve of breast cancer patients based on BTN1A1, BTN2A1, BTN2A2, BTN2A3, BTN3A1, BTN3A2, BTN3A3, BTNL2, BTNL3, and BTNL9 expression levels. Statistical difference (when log-rank *p* < 0.05) is shown. HR, hazard ratio.

Because of the facts that BTNs are reported to activate γδ T cells through specific TCR, and γδ T-cell number represents the best correlate of OS of a broad range of carcinomas, we analyzed correlation between the transcriptomic expression levels of BTN members with the prognosis of lung cancer and breast cancer patients. Kaplan–Meier OS survival curves of LUAD, LUSC, and breast cancer cases according to gene expression levels of BTN family members were plotted. Results showed that high expression of BTN1A1 (HR = 1.65, *p* = 2.1e-05), BTN2A3 (HR = 1.59, *p* = 9.8e-05), BTNL2 (HR = 1.33, *p* = 0.016), BTNL3 (HR = 1.9, *p* = 7.7e-08), and BTNL8 (HR = 1.32, *p* = 0.019) were significantly correlated with worse OS of LUAD (Figure 5A). However, low expressions of BTN2A1 (HR = 0.51, *p* = 6e-08), BTN2A2 (HR = 0.52, *p* = 7.2e-08), BTN3A1 (HR = 0.77, *p* = 0.032), BTN3A2 (HR = 0.51, *p* = 3.2e-08), BTN3A3 (HR = 0.48, *p* = 9.7e-10), and BTNL9 (HR=0.54, *p* = 9.1e-07) were significantly correlated with worse OS of LUAD (Figure 5A). Low expression of BTN3A3 (HR = 0.73, *p* = 0.0088) was significantly correlated with worse OS of LUSC (Figure 5B). These results suggested specific BTNs could serve as prognosis biomarkers for lung cancer. Because γδ T cells are involved in breast cancer (39, 40), we compared survival in high vs. low expression of BTN family in breast cancer and found low expressions of BTN1A1 (HR = 0.73, *p* = 1.3e-08), BTN2A1 (HR = 0.75, *p* = 3.7e-07), BTN2A2 (HR = 0.83, *p* = 0.00056), BTN2A3 (HR = 0.73, *p* = 2.2e-08), BTN3A1 (HR = 0.72, *p* = 2.3e-09), BTN3A2 (HR = 0.89, *p* = 0.042), BTN3A3 (HR = 0.86, *p* = 0.0064), BTNL2 (HR = 0.78, *p* = 8e-06), BTNL3 (HR = 0.71, *p* = 1.2e-09), and BTNL9 (HR = 0.62, *p* = 9.4e-10) were significantly correlated with worse OS of breast cancer (Figure 5C). These collectively showed that certain BTNs serve as biomarkers for cancer and potentially therapeutic targets.

## Discussion

There remain notable gaps in our knowledge of the function of BTNs in immune system, although several discoveries about the modulation in T cells were found (14). The receptor of each BTN member is not well-defined, making the identification of BTN ligands a priority (14). Meantime, several studies showed that the BTNs function before identifying the structural interaction with the unknown receptors. Phosphorantigens (IPP or BrHPP) could activate human γδ T cells, whereas the mechanisms remained unclear for a long time (14). Recently, Yang et al. (41) showed that BTN3A-pAg sensing mechanism involved and triggered the inside-out signaling to activate Vγ9Vδ2 T cells, using a structural approach. Moreover, BTN2A1 was found to directly bind to Vγ9Vδ2 TCR and involved in human γδ T-cell activation (42). These studies defined BTNs as important regulators of γδ T-cell function.

Both human and mouse γδ T cells have several subsets classified by their TCR chain, and each subset of γδ T cells plays distinct roles in many diseases (43). Our previous research in mice has proven that Vγ4 γδ T cells play a protective role in tumor immunity (44, 45), whereas Vγ1 γδ T cells suppress this function via interleukin 4 (IL-4) production (46). Also, our research indicated that Vγ4 γδ T cells play a protective role in the Con A–induced hepatitis through limiting natural killer T-cell activation by secretion of IL-17 (47). Collectively, these researches indicated different γδ T-cell subsets have intrinsic functional difference. However, how these γδ T-cell subsets were regulated remains unclear. Recent research has suggested specific BTN selects or shapes specific γδ T-cell subset in its specific location and time (17), and it is critical to further characterize the interactions between γδ T cells and BTNs. Based on the facts that γδ T cells have extraordinary capacity for tumor cell killing (43) and BTNs can activate γδ T cells (17), and structure of BTNs is closely sharing the fractions of B7 family, which plays a critical role in cancer immunity (4), we postulate BTNs play potential roles in cancer immunology and may serve as biomarkers for prognosis, although the accumulated publication amount of BTNs is much less than that of PD-1.

Through survival analysis of lung cancer and breast cancer patients with mRNA expression in gene chip data of BTNs, as we expected, several BTN members were proven as playing important roles in the prognosis of LUAD, LUSC, and breast cancer. Our results showed that high expressions of BTN1A1, BTN2A3, BTNL2, BTNL3, and BTNL8 were significantly correlated with worse OS of LUAD (Figure 5A). However, low expressions of BTN2A1, BTN2A2, BTN3A1, BTN3A2, BTN3A3, and BTNL9 were significantly correlated with worse OS of LUAD (Figure 5A). Low expression of BTN3A3 was significantly correlated with worse OS of LUSC (Figure 5B). In breast cancer, low expressions of BTN1A1, BTN2A1, BTN2A2, BTN2A3, BTN3A1, BTN3A2, BTN3A3, BTNL2, BTNL3, and BTNL9 were significantly correlated with worse OS (Figure 5C). These suggested a potential function of γδ T cells in cancer, which is regulated by BTNs. Currently, mechanisms of the effects are not clear; it is possible that different signal pathways are involved in specific immune microenvironment. With the impacts of Nobel Prize in 2018 (48), immune-checkpoint inhibition has been further utilized, and more researches have been done for the field. However, large amounts of people are unresponsive to immune therapy (27, 28). Based on previous research results, BTNs play important roles in cancer immunity, which might serve as new targets or therapeutic strategies and may be effective for anti–PD-1 or anti–cytotoxic T-lymphocyte-associated protein 4–unresponsive patients.

In summary, this research focuses on the immune regulation of BTNs and their correlation with the prognosis of lung cancer patients and provides a new aspect of gene therapy or immune intervention. Further efforts are still needed in investigation of the specific BTN receptor, mechanism of BTNs instructing local immune response (especially T-cell function), and the expression pattern of BTNs.

## Data Availability Statement

All datasets generated for this study are included in the article/Supplementary Material.

## Author Contributions

YWa and XZ did the bibiometric analysis and contributed to manuscript writing. NZ did patient survival calculation. ZLi, ZLia, JY, XLiu, YWu, KC, YG, ZY, XLin, and HZ designed the research and contributed to manuscript writing. DT, YC, and JH designed the research, organized the calculations and wrote the manuscript. All authors contributed to the article and approved the submitted version.

## Conflict of Interest

The authors declare that the research was conducted in the absence of any commercial or financial relationships that could be construed as a potential conflict of interest.
